# Structural Basis for Assembly of Hsp90-Sgt1-CHORD Protein Complexes: Implications for Chaperoning of NLR Innate Immunity Receptors

**DOI:** 10.1016/j.molcel.2010.05.010

**Published:** 2010-07-30

**Authors:** Minghao Zhang, Yasuhiro Kadota, Chrisostomos Prodromou, Ken Shirasu, Laurence H. Pearl

**Affiliations:** 1 Section of Structural Biology, The Institute of Cancer Research, Chester Beatty Laboratories, 237 Fulham Road, London SW3 6JB, UK; 2 RIKEN Plant Science Center, Tsurumi-ku, Yokohama 230-0045, Japan; 3 Genome Damage and Stability Centre, School of Life Sciences, University of Sussex, Falmer, Brighton BN1 9QH, UK

**Keywords:** PROTEINS, MOLIMMUNO

## Abstract

Hsp90-mediated function of NLR receptors in plant and animal innate immunity depends on the cochaperone Sgt1 and, at least in plants, on a cysteine- and histidine-rich domains (CHORD)-containing protein Rar1. Functionally, CHORD domains are associated with CS domains, either within the same protein, as in the mammalian melusin and Chp1, or in separate but interacting proteins, as in the plant Rar1 and Sgt1. Both CHORD and CS domains are independently capable of interacting with the molecular chaperone Hsp90 and can coexist in complexes with Hsp90. We have now determined the structure of an Hsp90-CS-CHORD ternary complex, providing a framework for understanding the dynamic nature of Hsp90-Rar1-Sgt1 complexes. Mutational and biochemical analyses define the architecture of the ternary complex that recruits nucleotide-binding leucine-rich repeat receptors (NLRs) by manipulating the structural elements to control the ATPase-dependent conformational cycle of the chaperone.

## Highlights

► Hsp90-N, Sgt1-CS, and Rar1-CHORD_II_ domains form a ternary complex ► The two CHORD domains of Rar1 have distinct properties ► CHORD domains interact with bound nucleotide in the N domain of Hsp90 ► Only CHORD_II_ can interact simultaneously with Sgt1 and Hsp90 and is essential for NLR recruitment

## Introduction

Cysteine and histidine-rich domains (CHORDs), are small zinc-containing domains, originally identified in the Rar1 protein that facilitates disease resistance (R) gene function in plants, and in the *C. elegans* Chp protein, whose ablation causes sterility and embryonic lethality (Shirasu et al., 1999). In all plant and animal CHORD proteins identified, the CHORD domains occur as tandem pairs, with a small zinc-binding cysteine-histidine C_3_H motif separating the two in the plant proteins (Heise et al., 2007). Animal CHORD proteins, Chp1 and melusin (Brancaccio et al., 2003b), possess an additional C-terminal CS domain, structurally related to small heat-shock proteins, α-crystallin and the Hsp90 cochaperone p23/Sba1 (Garcia-Ranea et al., 2002). Rar1 and melusin have been shown to interact with a second protein, Sgt1 (Azevedo et al., 2002; Sbroggio et al., 2008), which also possesses a CS domain, originally identified in budding yeast (Kitagawa et al., 1999), but with homologs in plants and animals. Rar1 and Sgt1 are essential for functioning of nucleotide binding leucine-rich repeat receptors (NLRs), encoded by disease resistance genes that mediate an innate immunity mechanism in plants (Shirasu and Schulze-Lefert, 2003). Mammalian Sgt1 is similarly essential for activation of NLRs, and mediates an innate immune response in animals (da Silva Correia et al., 2007; Mayor et al., 2007). While Rar1 is clearly involved in plant innate immunity, the involvement of mammalian CHORD proteins in NLR function is controversial, with conflicting observations (da Silva Correia et al., 2007; Hahn, 2005). No clear biological role has emerged for the ubiquitously expressed Chp-1. However, melusin, which is specifically expressed in the skeletal and cardiac muscles of vertebrates (Brancaccio et al., 1999), is directly implicated in the response to cardiac mechanical stress during pressure overload (Brancaccio et al., 2003a; De Acetis et al., 2005).

Sgt1's roles in plant innate immunity (in cooperation with Rar1), and probably all Sgt1 functions, are dependent on its interaction with the molecular chaperone, Hsp90 (Boter et al., 2007; Liu et al., 2004; Mayor et al., 2007; Shirasu, 2009; Zhang et al., 2008). Rar1 and the mammalian CHORD proteins are also independently able to bind Hsp90 (Sbroggio et al., 2008; Takahashi et al., 2003; Wu et al., 2005), as well as to Sgt1, so that stable Hsp90-Sgt1–CHORD protein complexes can be formed (Boter et al., 2007; Shirasu and Schulze-Lefert, 2003). The Crystallin and Small heat-shock protein like (CS) domain of Sgt1 plays a key role in these complexes, providing separate binding sites for Hsp90 (Catlett and Kaplan, 2006; Lee et al., 2004) and for Rar1 (Boter et al., 2007), while the SGT1-Specific (SGS) domain of Sgt1 provides the interaction with the leucine-rich repeat domain of plant and mammalian NLRs (Bieri et al., 2004; da Silva Correia et al., 2007; Leister et al., 2005). The role of the integral CS domain in mammalian CHORD proteins, absent from the plant Rar1, is unclear, and although not sufficient for interaction with Hsp90 on its own (Sbroggio et al., 2008), it may contribute to the stability of the overall interaction (Wu et al., 2005). Neither Sgt1 nor the CHORD domain proteins are themselves structurally dependent on Hsp90, suggesting that they are cochaperones rather than client proteins.

We have now determined the crystal structure of a ternary complex between the nucleotide binding N domain of Hsp90, the CS domain of Sgt1, and a CHORD domain of Rar1, providing a framework for understanding the architecture of Hsp90-Sgt1-CHORD protein complexes in plants and mammals. Mutational disruption of the observed interfaces results in defects in NLR protein-mediated viral resistance in plants, confirming the biological importance of the interactions. Structural and biochemical analysis defines the architecture of the Hsp90-Rar1-Sgt1 complex that recruits NLR clients, and suggest that CHORD domain proteins, like other Hsp90 cochaperones involved in client recruitment, manipulate the structural elements controlling the ATPase-coupled conformational cycle of the chaperone.

## Results

### Structure of Hsp90-N-Sgt1-CS-Rar1-CHORD_II_ Complex

**Figure 1:**
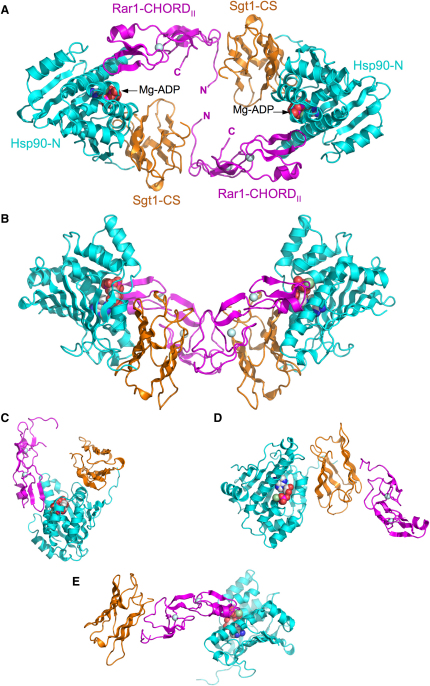
Crystal Structure of the H-S-R Complex (A) Hetero-hexameric assembly of Hsp90-N domain (cyan), Sgt1-CS domain (gold), and Rar1-CHORD_II_ domain (magenta). Mg^2+^-ADP bound in the nucleotide-binding pocket of the Hsp90 N domain is shown as a CPK model. The structural zinc atoms in the CHORD domain are shown as gray spheres. (B) As (A) but rotated 90° around the horizontal. (C) Ternary subcomplex centered on Hsp90-N. Sgt1-CS and Rar1-CHORD_II_ domains bind to nonoverlapping sites on Hsp90-N and make no direct mutual interaction. (D) As (C) but centered on Sgt1-CS. (E) As (D) but centered on Rar1-CHORD_II_.

The N-terminal domain of barley (*Hordeum vulgare*) Hsp90, the CS domain of cress (*Arabidopsis thaliana*) Sgt1a, and the CHORD_II_ domain of cress Rar1, were expressed, purified, and cocrystallized in the presence of ADP (see Experimental Procedures). The structure was solved by molecular replacement with the structure of the previously determined binary Hsp90-N-Sgt1-CS complex (Zhang et al., 2008), and refined at 2.2 Å resolution (see Experimental Procedures and Table 1). The overall architecture of the Hsp90-N-Sgt1-CS-Rar1-CHORD_II_ (H-S-R) complex is a puckered heterohexameric ring (Figures 1A and 1B) with approximate diad symmetry, and with the subunits in a cyclic a, b, c, a, b, c arrangement, so that each protein contacts only one copy of each of the other proteins (Figures 1C–1E). Although the cochaperones are from one plant species, and the Hsp90 from another, the amino acid sequences of the barley and cress homologs of the interacting domains of all three proteins are very high overall (Hsp90 ∼88% identity, Sgt1 ∼61% identity, Rar1 ∼68% identity), and the residues involved in the protein-protein interfaces are totally conserved.

**Figure 2:**
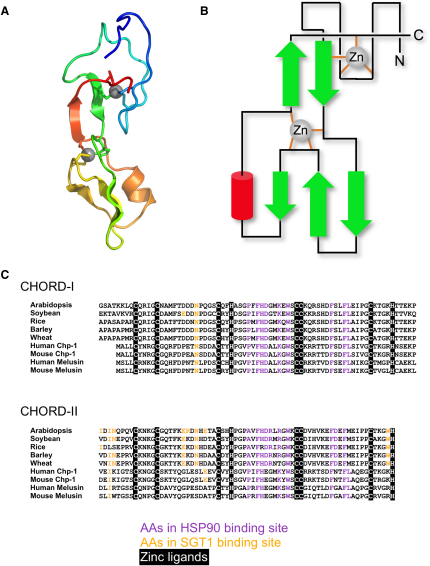
CHORD Domain Structure (A) Cartoon of the Rar1-CHORD_II_ domain rainbow colored N-blue → C-red. The structural zinc ions and their cysteine and histidine ligands, are indicated. (B) Schematic of CHORD domain fold. The overall structure is reminiscent of the Ring-finger domain, but the topological arrangement of the secondary structural elements is distinctive. (C) Amino acid sequence alignment of CHORD domains from various species. Black boxes highlight invariant amino acids in both CHORD_I_ and CHORD_II_ of all species, involved in liganding zinc ions. The important residues for Hsp90 interaction are shown in magenta and the residues for Sgt1 interaction are shown in orange.

The crystal structures of Hsp90-N and Sgt-CS have been described previously (Prodromou et al., 1997; Stebbins et al., 1997; Zhang et al., 2008), and their structures in the H-S-R complex are essentially identical. As expected (Heise et al., 2007; Shirasu et al., 1999), Rar1-CHORD is a metallo-protein domain with two structural Zn^2+^ ions (Figures 2A and 2B). The CHORD domain has a cylindrical structure ∼54 Å long and ∼13 Å in diameter. The C-terminal lobe consists of a three-stranded antiparallel β sheet with one face covered by a short α helix. The N-terminal lobe is devoid of secondary structure, apart from a short β strand, which makes an antiparallel interaction with a β strand extending from the C-terminal lobe, crossing back onto the N-terminal lobe. The C-terminal lobe chelates its zinc ion via the side chains of His181, and cysteines 196, 197, and 213. The N-terminal lobe binds zinc via cysteines 159, 164, and 178, and His218 from the returning C-terminal segment of the domain. The zinc-binding residues are totally conserved in all CHORD domains identifiable in plants and animals (Figure 2C).

### Hsp90-CS Domain Interactions

**Figure 3:**
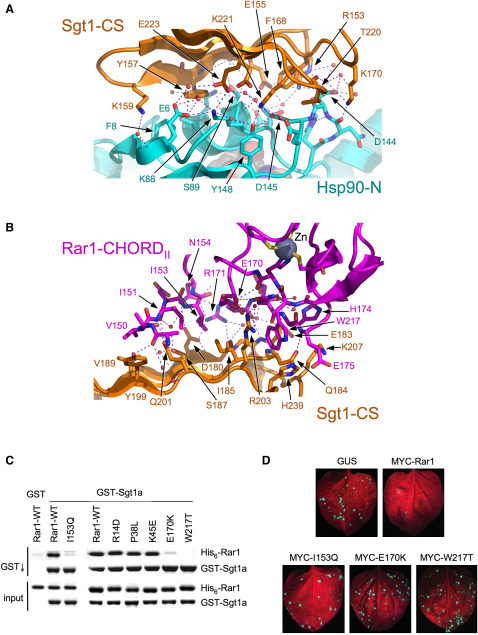
Sgt1 Interactions with Hsp90 and Rar1 (A) Detail of the interface between Sgt1-CS (gold) and Hsp90-N (cyan). The interface consists of two hydrophobic patches centered on Tyr 157 and Phe 168 of Sgt1-CS, supported by multiple hydrogen bonding and polar interactions (dashed lines). Mutation of interfacial residues (Tyr157, Phe168, Lys221, and Glu223) abrogates Hsp90-Sgt1 functional interaction (Zhang et al., 2008). (B) Detail of the interface between Sgt1-CS (gold) and Rar1-CHORD_II_ (magenta). The interacting residues in Rar1 are strongly conserved in the CHORD_II_, but not in the CHORD_I_ domains of plant CHORD proteins, or in animal CHORD domains. Mutation of Sgt1 residues Gly182 and Ile185 had previously been found to disrupt Sgt1 interaction with Rar1 (Boter et al., 2007). (C) Mutations of Rar1 residues Ile153, Glu170, or Trp217, which are all buried in the Rar1-CHORD_II_ interface with Sgt1-CS, disrupts interaction with Sgt1 in coprecipitation assays. (D) Mutation of Rar1 in Sgt1-interfacial residues abrogates the ability of transfected Rar1 to confer resistance to tobacco mosaic virus infection in Rar1-silenced tobacco leaves. GUS is transformed with the empty vector only.

Each subunit of the complex makes a distinct and nonoverlapping interaction with each of the other two subunits. Sgt1-CS interacts via the four-stranded face of its β sandwich structure, with Hsp90 residues from the N-terminal β strand 4-8, α helix 87-93, and the loop 141-148 connecting the fourth and fifth β strands (Figure 3A and Figure S1 available online). This interface is essentially identical to that previously described in the binary Hsp90-N-Sgt1-CS crystal structure (Zhang et al., 2008), and is not discussed further here.

There is no consensus as to the involvement of the CS domain of animal CHORD proteins, in interaction with Hsp90 (Sbroggio et al., 2008; Wu et al., 2005). To gain some insight into this, we mapped the amino acid sequences of the human Chp-1 and melusin CS domains onto the Sgt1 CS domain in the H-S-R complex described here, to determine whether residues involved in interaction with Hsp90 are conserved. For the key Sgt1-CS interfacial residues (Tyr157, Phe168, Lys221, and Glu223), whose mutation abrogates Hsp90-Sgt1 functional interaction (Zhang et al., 2008), the topological equivalents are, respectively, His, Tyr, Lys, and Glu in Chp-1 and His, Tyr, Arg, and Glu in melusin. None of these are incompatible with Hsp90 interactions made by Sgt1-CS, nor are there changes in the overall interface that would prevent the interaction. Thus, it is likely that the CS domain in vertebrate CHORD proteins is able to interact with Hsp90, but the affinity of that interaction remains to be determined.

### Sgt1-Rar1 Interaction

The three-stranded face of the Sgt1-CS structure, opposite the Hsp90-binding face (Figure 1D), provides the binding site for the Rar1-CHORD_II_ domain. One branch of the L-shaped interface centers on a hydrophobic interaction in which Sgt1:Ile185 packs into a recess formed by Rar1:Ile151, Ile153, and Glu170, with a peripheral contact between Sgt1:Tyr199 and Rar1:Val150 (Figure 3B). These interactions are reinforced by polar and solvent-bridged interactions between Sgt1:Tyr199, Gln201, Ser187, and Asp180, and the peptide backbone of Rar1:Ala148, Ile151, Ile153, and the side chain of Asn154, respectively.

The other branch of the interface involves polar interactions between the main and side chain of Rar1:Arg171 with the main chain of Sgt1:Phe181 and Glu183, and solvent-bridged and polar interactions between Rar1:Glu170, Asn173, and Sgt1:Arg203, and Rar1:Glu175 and Sgt1:Gln184, Lys207 and His239. This is anchored by Rar1:Trp217, whose indole ring makes a π-π stacking interaction with the guanidine of Sgt1:Arg203 and a proton-π interaction with the imidazole ring of His239.

All Rar1 residues involved are strongly conserved in plant CHORD_II_ domains, but not in plant CHORD_I_ or mammalian CHORD domains. This pattern of conservation is fully consistent with the observation that Rar1-CHORD_II_, but not Rar1-CHORD_I_, interacted with Sgt1-CS in a yeast two-hybrid assay (Azevedo et al., 2002). The affinity of the Rar1-CHORD_II_ interaction with Sgt1-CS, determined by isothermal titration calorimetry (ITC) was 3.09 (±0.15) μM (Figure S2C).

From the Rar1 side, mutations of Ile153, Glu170, or Trp217, substantially reduced interaction with Sgt1 in in vivo and in vitro analyses, confirming that the observed interface is authentic (Figure 3C, Figure S2). These mutations are all markedly defective in facilitating resistance to tobacco mosaic virus conferred by the corresponding NLR protein, N (Figure 3D). Thus, the Rar1-Sgt1 interaction does indeed play a key part in the essential role of these proteins in disease resistance.

As with the Hsp90 interaction, we analyzed the ability of the Chp-1 and melusin CS domain sequences, to mediate interaction with a CHORD domain. In contrast with the Hsp90 interaction, those involved in the CHORD interface are far less well conserved. In particular, Sgt1-CS residues Gly182 and Ile185, whose mutation abrogates interaction with Rar1 (Boter et al., 2007), are, respectively, Asn and Leu in Chp-1, and Ser and Glu in melusin—all incompatible with the observed Rar1-Sgt1-CS interface. With the absence of the N-terminal extension to the Rar1-CHORD_II_ domain in the vertebrate CHORD proteins, this makes it very unlikely that vertebrate CHORD-protein CS domains bind vertebrate CHORD domains.

### Rar1-Hsp90 Interactions

**Figure 4:**
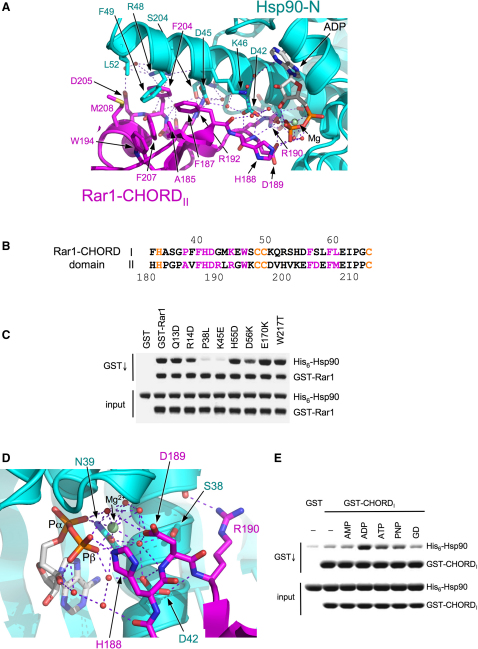
Rar1-Hsp90 Interactions (A) Detail of the interface between Rar1-CHORD_II_ (magenta) and Hsp90-N (cyan). The interface consists of a major hydrophobic patch on the CHORD domain, which interacts with a similar patch on the surface of the long helix in the Hsp90-N domain, reinforced with polar and solvent bridged interactions between the two proteins, and polar interactions between the CHORD domain and the nucleotide bound to Hsp90-N (see below). (B) Comparison of Hsp90-interacting residues in CHORD_I_ and CHORD_II_ domains. Residues involved in CHORD_II_ interaction with Hsp90 from the structure described here, and their equivalents in the CHORD_I_ domain, are shown in magenta. Zinc ligands are shown in gold. (C) Coprecipitation assays of Hsp90 with GST-Rar1 constructs bearing various point mutations in the CHORD_I_ domain. Mutations in residues outside the Hsp90-N interface predicted by homology with CHORD_II_, or in residues involved in Sgt1 interaction (Glu170 and Trp217), have no effect on the interaction. Bulky mutation of Pro38 which is predicted to be buried in the hydrophobic interface with Hsp90-N, or a charge reversal mutation of Lys45, which would participate in an ionic interaction with Asp45 of Hsp90-N, both diminish Rar1 interaction with Hsp90-N, confirming the primacy of CHORD_I_ in mediating the overall Rar1-Hsp90 complex. (D) Detail of the interactions between the Rar1 CHORD_II_ domain and the nucleotide bound to Hsp90-N. The imidazole ring of His188 interacts directly with the β-phosphate of the bound ADP, while the carboxylate side chain of Asp189 interacts with the salvation shell of the Mg^2+^ ion that bridges the α- and β-phosphate groups of the nucleotide. Mutation of either of these residues, which are very strongly conserved across all plant and animal CHORD domains, diminishes the interaction of Hsp90 and Rar1 in a yeast two-hybrid assay (Figure S1). (E) The interaction between the CHORD_I_ domain of Rar1 and Hsp90 is strongly influenced by the nature of the bound nucleotide. Consistent with the crystal structure, the presence of a β-phosphate and associated solvated Mg^2+^, as in ADP, ATP, or the nonhydrolyzable ATP analog AMP-PNP, significantly stabilize the Rar1-Hsp90 interaction, whereas AMP, or the inhibitor geldanamycin, do not. ATP-competitive inhibitors of Hsp90, such as geldanamycin, would most likely destabilize Hsp90-CHORD domain interactions in vivo.

Rar1 interacts with Hsp90 via the C-terminal lobe of the CHORD structure, at the opposite end to the Sgt1-interacting region. The core of the interface involves the interaction of Hsp90:Phe49 and Leu52 with a hydrophobic patch formed by the side chains of Rar1:Ala185, Phe187, Trp194, Phe204, Phe207, and Met208 (Figure 4A), augmented by polar and solvent bridged interactions involving Hsp90:Ser38, Ser41, Asp42, Asp45, Lys46, Arg48, and Ser200, and Rar1:Arg190, Arg192, Glu203, and Asp205. All Hsp90 residues involved are invariant, with the exception of Phe49, which is tyrosine in nonplant species, and fully compatible with the observed interface. All Rar1 residues involved are also well conserved, with key hydrophobic residues essentially invariant in both CHORD domains from all sources (Figure 4B), suggesting that the ability to bind Hsp90-N is a conserved feature of all CHORDs, unlike the interaction with Sgt1, which is a unique property of plant CHORD_II_. While CHORD_II_ can bind Hsp90-N, its interaction in isolation is weaker than that of CHORD_I_, and mutations targeting the Hsp90-interacting residues on CHORD_I_ have a more significant effect on the Rar1-Hsp90 interaction (Figures 4C and S3), suggesting that the Hsp90-N-CHORD_I_ interaction makes the major contribution to the binary Hsp90-Rar1 complex.

Peripheral to the main interface, the side chains of two Rar1 residues interact directly with the Mg-ADP bound in the nucleotide pocket of the Hsp90 N domain (Figure 4D). The side chain of His188 hydrogen bonds to the β-phosphate of the ADP, while the side chain of Asp189 interacts with two water molecules attached to the magnesium ion bound between the ADP phosphates and Hsp90:Asn39. The conformation of the loop carrying His188 and Asp189 is stabilized by Hsp90:Asp42, which accepts hydrogen bonds from the backbone amide groups of Asp189 and Arg190. Despite Rar1:His188 and Asp189 being exposed polar residues at the tip of a loop with no side-chain contacts with the rest of the domain, they are highly conserved in all CHORD domain sequences, with His188 invariant and Asp189 conservatively varied to glutamate in some examples. To determine whether nucleotide loading of the Hsp90 N domain is significant in the interaction, we looked at the ability of GST-CHORD_I_ to pull down Hsp90 in the absence of nucleotide or in the presence of different nucleotides and nucleotide mimetics. Although apo-Hsp90 was coprecipitated by GST-CHORD_I_ more strongly than with GST alone, the interaction was significantly enhanced by addition of ADP, and to a lesser degree AMP, ATP, or the nonhydrolyzable ATP analog AMP-PNP (Figure 4E), which provide most of the interactions observed in the crystal structure with Rar1:His188 and Asp189. The Hsp90 inhibitor geldanamycin, although filling the ATP-binding site, provides none of the observed interactions, and doesn't enhance CHORD domain binding.

### Dynamic Architecture of Hsp90-CS-CHORD Complexes

**Figure 5:**
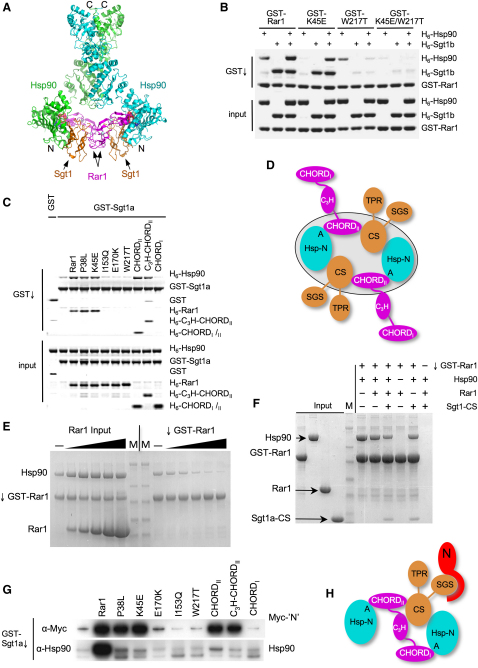
Formation of the Ternary Hsp90-Sgt1-Rar1 Complex (A) Model of full-length Hsp90 complex with Sgt1-CS and Rar1-CHORD domains. This was constructed by the simultaneous least-squares superposition of the two Hsp90-N domain in the crystal structure described here, onto the N domains in the structure of AMPPNP-bound Hsp90 in complex with p23/Sba1 (Ali et al., 2006). Flexibility between the C-, middle, and N domains of Hsp90 permits accommodation of the Sgt1-CS-Rar1-CHORD subcomplex as a bridge between the two N domains, and would allow productive engagement of the middle and N domains in a catalytically active conformation. (B) Coprecipitation of Hsp90 and Sgt1, by Rar1. Wild-type GST-Rar1 coprecipitates Hsp90 or Sgt1 individually, but the interaction with Hsp90 in particular is strengthened by the presence of Sgt1. A mutation of Rar1 (K45E) that impairs interaction with Hsp90 in the absence of Sgt1, is able to interact with Hsp90 in the presence of Sgt1 and establish a stable ternary complex. A Rar1 mutant that cannot interact productively with Sgt1 (W217T), binds Hsp90 in the absence of Sgt1, but its interaction with Hsp90 is greatly diminished when Sgt1 is present, suggesting steric interference between Sgt1 and Rar1 in their individual interactions with Hsp90, when they are unable to form a mutual bridging interaction. The Rar1 double mutant abolishes all interaction with Hsp90 or Sgt1, direct or bridged. (C) The CHORD_II_ domain of Rar1 is necessary and sufficient to form a ternary complex. Consistent with (B) GST-Sgt1 will coprecipitate Hsp90 and wild-type Rar1, or Rar1 with mutations in the direct Hsp90 interface on CHORD_I_ (P38L, K45E), but not with Rar1 mutations in the CHORD_II_ binding site for Sgt1 (I153Q, E170K, W217T), in a parallel to the interference effect observed in (B). Isolated CHORD_II_ or a construct of CHORD_II_ and the inter-CHORD linker, fully recapitulate formation of a stable ternary complex. CHORD_II_ mutants that cannot bind Sgt1 compete with Sgt1 for binding to HSP90. (D) A putative symmetrical arrangement for an Hsp90-Sgt1-Rar1 complex, based on the crystal structure presented here, in which the CHORD_II_ domain participates in the formation of a heterohexameric “ring” complex, reinforced by the mutual scaffolding of the three pairwise interfaces. (E) Titration of increasing amounts of untagged Rar1 progressively competes out the amount of Hsp90 that can be coprecipitated by GST-tagged Rar1. However, no untagged Rar1 is coprecipitated, showing that the Hsp90 dimer cannot simultaneously bind two Rar1 molecules. (F) When Sgt1-CS domain is present, it can be coprecipitated along with Hsp90 by the GST-tagged Rar1, however the presence of Sgt1-CS does not facilitate an Hsp90 dimer-bridged interaction with a second Rar1 molecule. (G) GST-tagged Sgt1 coprecipitation of Hsp90 and Myc-tagged disease resistance protein N (from *Nicotiana tabaccum*), in the presence of Rar1 constructs/mutants, in a wheat-germ cell-free lysate. In the presence of wild-type Rar1, Sgt1 efficiently coprecipitated Hsp90 and Myc-N. With Rar1 harboring mutations in residues involved in the CHORD_I_ interface with Hsp90 (P38L, K45E), Sgt1 coprecipitation of Myc-N was retained, however Hsp90 binding was reduced, but not eliminated. Mutations in Rar1 residues in the CHORD_II_ interface with Sgt1-CS domain (E170K, I153Q, W217T), greatly reduced binding of Hsp90 and Myc-N protein, confirming to cooperation of Rar1 and Sgt1 in recruitment of clients to the chaperone system. The function of Rar1 in facilitating interaction of Sgt1 with Myc-N, could be fully restored by truncated Rar1 mutants lacking the CHORD_I_ domain that mediates tight binding to Hsp90. (H) Model of an asymmetric Hsp90-Sgt1-Rar1 complex based on the crystal structure presented here, and fully consistent with the biochemical interaction data. A single Rar1 molecule occupies both nucleotide-bound Hsp90 N domain binding sites, by interaction of the conserved motifs in CHORD_I_ and CHORD_II_ domains. The CHORD_II_ domain bridges one Hsp90 N domain to the other via interaction with the Sgt1 CSD domain. The CHORD_II_-CS interaction stabilizes binding of a client LRR-NB protein such as N, to the SGS domain of Sgt1—the mechanistic basis for this is not yet understood.

Comparison with structures of dimeric full-length Hsp90 (Ali et al., 2006), or related GRP94 (Dollins et al., 2007) and bacterial HtpG (Shiau et al., 2006), shows the distance between the two Hsp90-N domains in the H-S-R complex and their relative orientation, is compatible with them being part of an intact Hsp90 dimer (Figure 5A). The H-S-R complex as crystallized contains two copies of the Rar1-CHORD_II_ domain each bound to one of the Hsp90-N domains and to an Sgt1-CS domain, which itself interacts with a nonoverlapping site on Hsp90-N. Thus, each protein interacts simultaneously with the other two, generating a cooperative and self-supporting assembly, which is a stable species in solution (Figure S4). Consistent with this we find that Sgt1 reinforces the interaction of Rar1 with Hsp90, and the three proteins form a stable ternary complex (Figure 5B).

The ternary complex is stable to a mutation in the Rar1-CHORD_I_ (Lys45Glu) that severely reduces Rar1 binding to Hsp90 in the absence of Sgt1, but not to a Rar1 mutation (Trp217Thr) that abrogates the Rar1-CHORD_II_-Sgt1-CS interaction. Although in isolation CHORD_II_ binds Hsp90 more weakly than CHORD_I_, it alone can stabilize the ternary complex as well as the full-length Rar1 protein, but not in the presence of mutations that abrogate the Rar1-Sgt1 interaction (Figures 5B and 5C). Thus, in the presence of Sgt1, the configuration observed in the crystal structure, with the Rar1-CHORD_I_ domain and the Sgt1-Tetratricopeptide Repeat (TPR) and SGS domains (and putative bound Nucleotide-Binding Leucine Rich Repeat [NB-LRR] client protein), disengaged from the core, is certainly stable (Figure 5D).

In the absence of Sgt1, the situation is reversed. Thus, while mutations in CHORD_II_ residues implicated in interaction with Hsp90 have a small effect on Rar1 binding, mutation of the equivalent residues in Rar1-CHORD_I_ (Lys45Glu, Pro38Leu) greatly diminish the binary Rar1-Hsp90 interaction (Figure 4C). While CHORD_I_ binding to Hsp90-N is inherently stronger than CHORD_II_, with CHORD_I_ of a Rar1 molecule bound to one of the N domains in an Hsp90 dimer, the effective concentration of the attached CHORD_II_ domain will be greatly enhanced, facilitating its binding to the other N domain, so that a single Rar1 molecule could in principle bridge the N domains of an Hsp90 dimer. The residues between the C terminus of the first CHORD domain (76) and the N terminus of the second CHORD domain (148), would readily span the observed distance of ∼18 Å between the visible N and C termini of the CHORD domains in the crystal structure (Figure 1A).

To determine if a single Rar1 molecule can occupy both N-terminal binding sites within an Hsp90 dimer, we used GST-Rar1 to coprecipitate Hsp90 in the presence or absence of untagged Rar1. The amount of Hsp90 pulled down by GST-Rar1 was progressively diminished by addition of increasing amounts of untagged Rar1, suggesting direct competition between the GST-tagged and untagged Rar1, and with no untagged Rar1 coprecipitated as would be expected if each Rar1 molecule only occupied one of the two CHORD domain binding sites furnished by the Hsp90 dimer (Figure 5E). Similar results were obtained when isolated CHORD_I_ was added in competition (Figure S4C). Addition of Sgt1-CS did not promote coprecipitation of untagged Rar1, but was itself coprecipitated by GST-Rar1 in the presence of Hsp90, consistent with formation of a ternary complex (Figure 5F).

Together, these data suggest a mechanism for chaperone recruitment of Sgt1/Rar1-dependent recruitment of NLR innate immunity proteins. Thus initial interaction of Rar1 via its CHORD_I_ domain, facilitates recruitment of Sgt1, whose simultaneous binding to Hsp90 gives the stable CHORD_II_-mediated Hsp90-Sgt1-Rar1 ternary interaction observed in the crystals. The functional importance of CHORD_II_ and its interaction with Sgt1-CS is underlined by the observation that alone, it can facilitate recruitment of an NLR client protein to Sgt1, to a similar degree to the intact Rar1 protein, or to a Rar1 protein containing CHORD_I_ mutations that disrupt direct interactions with Hsp90 (Figure 5G). This ability is lost in the context of Rar1 with mutations that disrupt CHORD_II_ interaction with Sgt1-CS. However, stable association of Hsp90 with a Rar1-Sgt1-NLR complex requires both CHORD domains, deployed in an asymmetric arrangement with Sgt1, which thereby delivers a single NLR client to the Hsp90 chaperone system (Figure 5H, Figure S4).

### CHORD Domain Manipulation of Hsp90 ATPase Mechanism

Several Hsp90 cochaperones directly influence the conformationally coupled ATPase mechanism of the chaperone, and/or bind preferentially to specific nucleotide-loaded states (Ali et al., 2006; Meyer et al., 2004; Panaretou et al., 2002; Prodromou et al., 1999; Roe et al., 2004; Siligardi et al., 2002, 2004; Sullivan et al., 1997). Sgt1 preferentially binds Hsp90 in the absence of ATP, but its bound position on the N domain of the chaperone, distant from the nucleotide-binding pocket (Zhang et al., 2008), does not affect any of the conformational switches that occur as part of the ATPase-coupled chaperone cycle (Ali et al., 2006; Siligardi et al., 2004; Vaughan et al., 2009), nor influence the ATPase activity (Boter et al., 2007; Catlett and Kaplan, 2006; Lee et al., 2004).

**Figure 6:**
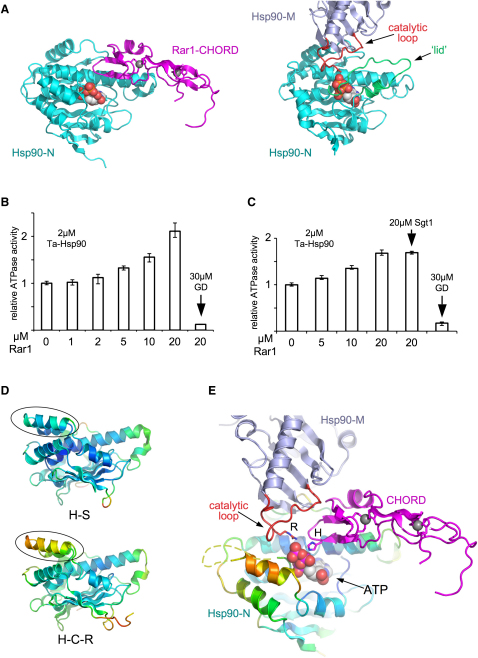
CHORD Domain Binding and ATPase Activity (A) Comparison of the Hsp90-N-Rar1-CHORD interaction (left) and the intramolecular interaction of the middle (blue gray) and N domain of Hsp90 in the catalytically active ATP-bound conformation. The middle domain catalytic loop bearing Arg380 (in yeast Hsp90) is shown in red, and the closed conformation of the “lid” segment is shown in green. The position of the bound CHORD domain (magenta) overlaps the position of the lid in the closed conformation, so that the two are mutually exclusive. (B) Effect of Rar1 on the inherent ATPase of Hsp90. Increasing concentrations of Rar1 promote and increase in the ATP turnover of Ta-Hsp90, achieving a 2-fold increase at 20-fold excess. ATP hydrolysis is fully inhibited by the Hsp90-specific inhibitor geldanamycin (GD), demonstrating that the observed ATPase activity is a function of Hsp90 itself. Error bars are SD of triplicate measurements. (C) Addition of Sgt1 at the same concentration of Rar1, has no effect on the ability of Rar1 to stimulate Hsp90s ATPase activity. Error bars are SD of triplicate measurements. (D) Rar1 binding promotes an order-disorder transition in the lid segment of the Hsp90 N domain. The lid segment in Hsp90-N domain from the binary Hsp90-N-Sgt1-CS complex (H-S) (Zhang et al., 2008), is well ordered and shows relative thermal parameters (rainbow colored low: blue → high: red) comparable to the core structure of the domain. In the ternary complex (H-S-R), the lid displays significantly higher thermal parameters, and in some crystals is fully disordered, with little coherent electron density for much of its structure. (E) Model for a catalytically productive Hsp90-CHORD domain complex, constructed by superposition of the N domain from full-length ATP-bound Hsp90 (Ali et al., 2006) on the equivalent domain in the Hsp90-Sgt1-Rar1 complex described here. Binding of the CHORD domain promotes disordering and displacement of the lid segment (orange dashed line), allowing access of the loop bearing the catalytic arginine residue from the middle domain, to the bound ATP. The conserved histidine of the CHORD domain interacts with the β-phosphate of the ATP, replacing the interaction provided by the peptide NH groups at the hinge of the lid. The CHORD domain would be positioned to interact with and stabilize the catalytic loop in its active conformation, thereby promoting catalysis.

The CHORD domain interaction with the nucleotide-binding pocket of Hsp90, and indeed with the nucleotide itself, would be expected to have some direct effect on ATPase activity. Superposition of the Hsp90-N in the Hsp90-N-Rar1-CHORD binary complex onto the structure of full-length Hsp90 in the ATP-bound conformation (Ali et al., 2006), reveals a significant steric overlap between the CHORD domain in the Rar1-Hsp90 complex, and the N domain “lid” segment in the closed conformation in ATP-bound Hsp90, that permits access by Arg380 on the middle domain catalytic loop to the γ-phosphate of ATP (Figure 6A).

Thus, Rar1 binding and the Hsp90 conformation required for ATP hydrolysis appear to be mutually exclusive, and at first sight Rar1 would be expected to inhibit the ATPase activity. Surprisingly, addition of Rar1 did not inhibit Hsp90's ATPase activity, but actually elicited a small increase in basal ATP turnover (Figure 6B). Comparable activation was observed when Sgt1 was present in addition to Rar1 (Figures 6C and S5). Rar1 itself displayed no inherent ATPase activity (data not shown). Mutation of the catalytic arginine residue in the middle domain of Hsp90 completely abolished ATPase activity in the absence or presence of Rar1, confirming that the Rar1-stimulated activity was an inherent property of Hsp90 itself, and that it depended on assembly of the split ATPase active site of the chaperone, as previously described (Ali et al., 2006).

Examination of the superimposed Hsp90-N-Rar1-CHORD binary complex and full-length ATP-bound Hsp90 structures, confirms that the CHORD domain presents no steric obstacle to productive docking of the middle domain of the chaperone onto the N domain. So long as the lid segment is displaced from its open conformation, access of the Hsp90 catalytic arginine to the nucleotide-binding pocket, is unimpeded by a bound CHORD domain. In fact, the lid segment in the H-S-R complex has high temperature factors relative to the rest of the Hsp90-N domain, and in some crystals, is substantially disordered (Figure 6D). Disordered lid segments occur in several Hsp90 crystal structures from different biological sources (Dollins et al., 2007; Huai et al., 2005) and are by no means unusual. By contrast, the equivalent segment in the Hsp90-N-Sgt1-CS binary complex (Zhang et al., 2008) is well ordered with low relative temperature factors. Thus, CHORD domain binding facilitates an order-disorder transition in the lid segment that facilitates its displacement and permits access by the middle domain catalytic loop. Furthermore, the N-terminal side of the catalytic loop in its active conformation, would interact with the surface of the bound CHORD domain. The Hsp90 activator Aha1 (Panaretou et al., 2002), achieves its effect by interacting with the C-terminal side of the Hsp90 catalytic loop, and facilitating its transition to an active conformation (Meyer et al., 2003, 2004). The location of the CHORD domain bound to Hsp90-N, suggests that it too might facilitate the ATPase activity of Hsp90 by stabilizing the active conformation of the catalytic loop (Figure 6E).

## Discussion

The involvement of Hsp90 in establishing the function of plant NLR disease resistance proteins is well established (Hubert et al., 2003; Liu et al., 2004; Lu et al., 2003; Takahashi et al., 2003; Zhang et al., 2004). Hsp90 has also been implicated in the regulation and activation of Nod-like receptors (da Silva Correia et al., 2007; Mayor et al., 2007), the mammalian orthologs of the plant disease resistance proteins, revealing a conserved Hsp90-dependent mechanism of innate immunity in animals and plants (Shirasu, 2009; Ye and Ting, 2008).

The essential involvement of the Hsp90 cochaperone Sgt1, in the function of these NLR is also clear (Mayor et al., 2007; Shirasu, 2009; Ye and Ting, 2008), but only in plants has the requirement for an additional CHORD cochaperone, Rar1, been clearly established (Azevedo et al., 2002; Bieri et al., 2004; Liu et al., 2004; Muskett et al., 2002; Shirasu et al., 1999). Both mammalian CHORD proteins, Chp1 and melusin, have been shown to bind Hsp90 (Sbroggio et al., 2008; Wu et al., 2005) and the latter also to mammalian Sgt1. However, the residues involved in the plant Rar1-CHORD-Sgt1-CS interaction are not conserved in mammalian melusin and Sgt1 proteins, so a different mode of interaction of the two proteins may be involved. Whether melusin and mammalian Sgt1 can bind Hsp90 simultaneously, as do their plant counterparts, remains to be determined.

Sgt1 acts as a client-specific adaptor, binding Hsp90 via its CS domain, and the leucine-rich repeat domain of clients such as NLR innate immunity receptors in plants and animals, or the adenylyl cyclase Cyr1p/Cdc35p in budding yeast (Dubacq et al., 2002). Unlike other client adaptors such as Cdc37 or Hop/Sti1 (Prodromou et al., 1999; Siligardi et al., 2002), Sgt1 has no inherent Hsp90 ATPase regulatory activity (Catlett and Kaplan, 2006; Lee et al., 2004; Zhang et al., 2008), and the small ATPase activation observed with Rar1 binding is unlikely to be significant. Sgt1-Rar1 binding to Hsp90 does, however, have the important effect of decoupling ATP turnover from the N domain dimerization and lid closure stabilized by the cochaperone p23/Sba1 (Ali et al., 2006), and permits a less constrained “transition state” for the chaperone ATPase cycle. What specific role this plays in regulation or activation of NLR receptors is not known.

Plant and mammalian NLRs constitute a conserved mechanism of innate immunity, dependent on Hsp90 and Sgt1 (Shirasu, 2009). While Rar1 is firmly implicated in this mechanism in plants, a role for the mammalian CHORD proteins in innate immunity has not been clearly established. Within the plant system, Sgt1 provides multiple binding functions, interacting simultaneously with Hsp90 and Rar1 via opposite faces of its CS domain, and the NLR receptor (Figure 5H), via its C-terminal SGS domain (Bieri et al., 2004; da Silva Correia et al., 2007; Leister et al., 2005). Sgt1 can also simultaneously bind Hsp90 and the Skp1p-Cdc53p-F box (SCF) E3 ubiquitin ligase subunit Skp1 via its TPR domain (Catlett and Kaplan, 2006; Kadota et al., 2008), and in budding yeast *sgt1* mutant strains are defective in targeted ubiquitination (Kitagawa et al., 1999). The multiple interactions of Sgt1 raise the possibility that it facilitates a functional interaction between innate immunity receptors and E3 ubiquitin ligases that may contribute to the activity of those receptors. Formation of ubiquitin chains is well described in the regulation of membrane-associated innate immunity receptors of the Toll-like family (Lowe et al., 2006), and there are indications that ubiquitination via SCF complexes plays a role in the function of plant NLR proteins dependent on Hsp90 and Sgt1 (Liu et al., 2002). Whether involvement of the ubiquitination machinery is a common feature of Hsp90-Sgt1-dependent plant disease resistance systems, and whether this extends to the mammalian systems, is unclear.

## Experimental Procedures

### Protein Expression and Site-Directed Mutagenesis

Plasmid constructs for recombinant protein expression of Hsp90 (*Arabidopsis*, *H. vulgare*, and *T. aestivum*), Sgt1a, Sgt1b, and CS (*Arabidopsis*) were as described previously (Boter et al., 2007; Kadota et al., 2008; Zhang et al., 2008). Rar1 mutants were obtained by site-directed mutagenesis (GeneTailor, Invitrogen). The cDNA of *N* was fused to Myc-tag sequence at the C-terminal end and cloned into pF3K, an expression vector for the wheat-germ cell-free system (Promega), and expressed according to the manufacturer's instructions.

### Crystallization, Data Collection, and Structure Determination

*H. vulgare* Hsp90 N domain, *Arabidopsis* Sgt1a CS domain, and Rar1 CHORD_II_ domain were combined in a 1:1:1 molar ratio with 5 mM ADP, incubated for 30 min, and concentrated to 10 mg/ml by ultrafiltration. Crystals were grown by vapor diffusion at 20°C against 100 mM HEPES (pH 7.5) and 23% w/v PEG5000 monomethylether (MME), and harvested in reservoir solution with glycerol (20% v/v) before cooling to 100 K. X-ray data were collected on beamline I03 at the Diamond Light Source (Didcot UK) from single crystals, and processed with MOSFLM (Leslie, 1995) and the CCP4 package (CCP4, 1994). Crystals had space group *P32* with two copies of the complex in the asymmetric unit. The structure was solved by molecular replacement with Protein Data Bank (PDB) entry 2JKI using Phaser (CCP4, 1994). Structures were built into difference Fourier density with COOT (Emsley and Cowtan, 2004) and refined with Phenix (Adams et al., 2002). Structure factors and refined coordinates have been deposited in the PDB as 2XCM. Crystallographic statistics are in Table 1.

### Y2H Analysis

*SGT1a* and *SGT1b* were cloned in pB42AD (Clontech). HSP90 (*Arabidopsis* and *H. vulgare*) and its derivatives in pB42AD were described earlier (Boter et al., 2007; Takahashi et al., 2003). Interaction analyses were carried out as described in the manufacturer's protocol (MATCHMAKER; Clontech).

### Plant Materials, VIGS Experiments, Transient Expression, and Resistance Assay

The VIGS experiments and the transient expression system using transgenic *N. benthamiana* plants expressing *N* under the control of its own promoter were described previously (Azevedo et al., 2006; Liu et al., 2002). *Agrobacterium* expressing *Arabidopsis Rar1* or its derivates (OD_600_ = 0.3) was infiltrated into leaves and 3 days after the first inoculation, *Agrobacterium* carrying *TMV-GFP* is infiltrated again in the same location (OD_600_ = 0.01). TMV accumulation was monitored by GFP fluorescence under UV illumination 4 days after the second inoculation.

### Coimmunoprecipitation Assay

*Agrobacterium*-infiltrated *N. benthamiana* leaves were crosslinked by formaldehyde as described (Rohila et al., 2004). Leaf tissues (0.5 g) were grounded to a powder in liquid nitrogen and resuspended in 2.5 ml of the extraction buffer (25 mM Tris-HCl pH 7.5, 100 mM NaCl, 2.5 mM MgCl_2_, 10% glycerol, 5 mM dithiothreitol (DTT), 50 μM ZnCl_2_, 20 mM MoO_4_) with the protease inhibitor cocktail (Roche) and 2% polyvinylpolypyrrolidone. The extracts were spun down at 15,000 g at 4°C for 10 min at least for three times. The supernatant (2 ml) in a total volume of 10 ml of the extraction buffer with 0.15% Nonidet P-40 (IP buffer) was added to α-myc (9E10) agarose beads (Santa Cruz). The mixture was incubated at 4°C for 1 hr, washed four times with IP buffer, and the pellet was resuspended in 1× SDS-PAGE loading buffer. Western blotting was performed with α-HA antibody (3F10 Roche), α-Myc antibody (A14, Santa Cruz), and α-HSP90 antibody (Kadota et al., 2008).

### ATPase Assay of HSP90

ATPase activity of Hsp90 was performed as previously described (Panaretou et al., 1998).

Other experimental details are given in the Supplemental Information.

## Figures and Tables

**Table 1:** Crystallographic Statistics

Data Collection	
Space group	P3_2_
Cell dimensions a, c (Å)	88.89, 117.820
Resolution (Å)	46.8–2.2
R_merge_	0.062 (0.285)
I/σ(I)	9.2 (2.2)
Completeness (%)	99.3 (99.5)
Redundancy	3.1 (3.1)

**Refinement**	

Resolution (Å)	46.8–2.2
No. reflections	52,455
R_work_/R_free_	19.82/24.19
No. atoms: protein	6004
Ligand	56
Solvent	608
<B factors>: protein + ligand	32.33
Solvent	37.98
Rmsd: bond lengths (Å)	0.008
Bond angles (°)	1.219
Ramachandran plot: % favorable + allowed	98.5
Ramachandran plot: % unfavorable	1.5

Statistics in parentheses refer to outer resolution shell.

## Accession Numbers

Experimental structure factors and refined coordinates for the Hsp90-Sgt1-Rar1 complex described here have been deposited in the Protein Data Bank under ID 2xcm.

## Supplemental Information

Document S1. Supplemental Experimental Procedures and Five Figures
