# Supplementary material for: Structural Basis for Assembly of Hsp90-Sgt1-CHORD Protein Complexes: Implications for Chaperoning of NLR Innate Immunity Receptors
Source: Mol Cell. 2010 Jul 30;39(2):269–81. doi: 10.1016/j.molcel.2010.05.010 (PMC2935968; doi:10.1016/j.molcel.2010.05.010)
Supplement: Document S1. Supplemental Experimental Procedures and Five Figures [file mmc1.pdf]

Molecular Cell, ***Volume 39***

Supplemental Information

Structural Basis for Assembly of Hsp90-Sgt1-CHORD Protein Complexes: Implications for Chaperoning of NLR Innate Immunity Receptors

Minghao Zhang, Yasuhiro Kadota, Chrisostomos Prodromou, Ken Shirasu, and Laurence H. Pearl

Supplemental Experimental Procedures

Isothermal Titration Calorimetry and Kd Determinations

Heat of interaction was measured on an ITC_{200} microcalorimeter (Microcal), with a cell volume of 200 μl, under the same buffer conditions (20 mM Tris pH 7.5 containing 1 mM EDTA and 5 mM NaCl). A) 10 aliquots of 3.8 μl of 500 μM N-terminal Hv-Hsp90 were injected into 50 μM Chord II domain of At-Rar1 at 30°C. B), 10 aliquots of 3.8 μl of 400 μM Chord II domain of At-Rar1 were injected into 35 μM Cs-domain of At-Sgt1a at 10°C. Heats of dilution were determined in a separate experiment by diluting protein into buffer, and the corrected data fitted using a nonlinear least square curve-fitting algorithm (Microcal Origin) with three floating variables: stoichiometry, binding constant and change in enthalpy of interaction.

```
                    157        168             182   185
At SGT1A        ---EYYQKPEEVVVTVFAKGIP--KQNVNIDFG---EQILSVVIEVP-GEDAYYLQPRLF 205
Hs Sgt1         ---DWYQTESQVVITLMIKNVQ--KNDVNVEFS---EKELSALVKLP-SGEDYNLKLELL
Hs melusin      CRHDWHQTDSLVVVTVYGQIPL--PAFNWVKAS---QTELHVHIVFD-GNRVFQAQMKLW
Hs CHP-1        CRHDWHQTGGEVTISVYAKNSL--PELSRVEAN---STLLNVHIVFE-GEKEFDQNVKLW
Sc SGT1         ---DWYQSSTSVTISLFTVNLPESKEQVNIYISPNDRRTLSISYQVPKSGSEFQYNAKLS
                   :::*.   *.:::             :  .      *     .  .   :  : .*

                              221
At SGT1A        GKIIPDKCKYEVLSTKIEICLAKADIITWASLE 238
Hs Sgt1         HPIIPEQSTFKVLSTKIEIKLKKPEAVRWEKLE
Hs melusin      GVINVEQSSVFLMPSRVEISLVKADPGSWAQLE
Hs CHP-1        GVIDVKRSYVTMTATKIEITMRKAEPMQWASLE
Sc SGT1         HEVDPKAVSLKIFPKKLEITLSKIDSTQWKKLE
                  :  .     :  ..::** : * :   * .**
```

Figure S1 – (related to Figure 3) - Amino acid conservation in CS domains

Alignment of CS domain sequences from A.thalania, S.cerevisiae and H.sapiens Sgt1 proteins, with those from the mammalian CHORD-proteins melusin and Chp-1. Residues that play a key role in the experimentally characterised interaction of AtSgt1 and Hsp90 are highlighted in yellow. The conservation of these in the CS domains of the mammalian CHORD proteins, suggests that these are able to interact with Hsp90 in a similar fashion. Residues in the plant Sgt1-CS domain involved in interaction with the Rar1 $CHORD_{II}$ are highlighted in pink – these are poorly conserved in the mammalian CHORD protein CS domains and in the mammalian Sgt1 domains.

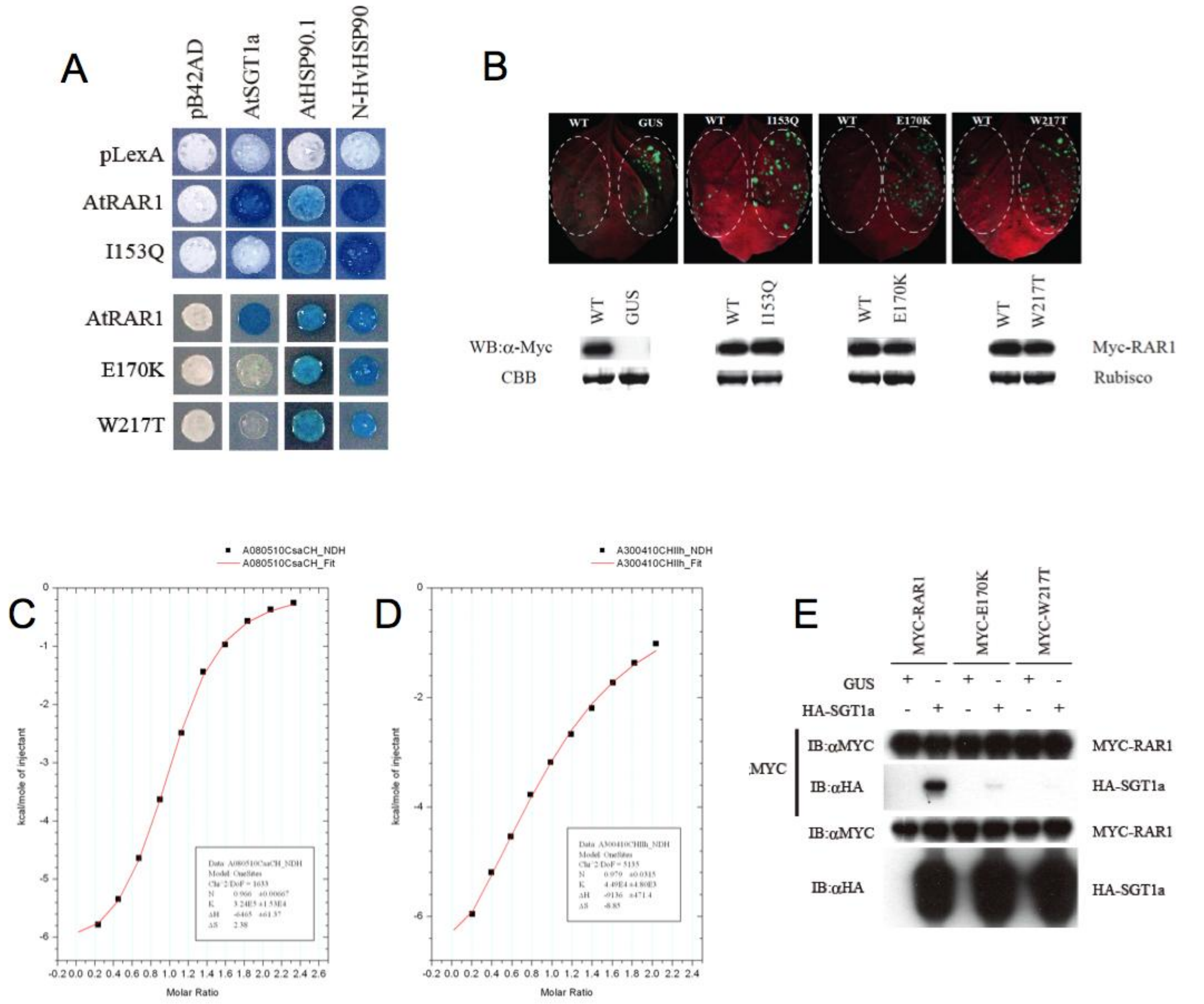

Figure S2 – (related to Figure 3) - Sgt1 Interactions with Hsp90 and Rar1

A) Mutation of Rar1 residues, I153, E170 or W217, disrupts interaction with Sgt1 in yeast two hybrid assay and *in vivo* co-immunoprecipitation assay B).

C) Isothermal titration calorimetry (ITC) of 400μM Rar1-CHORD$_{II}$ injected into 35μM Sgt1-CS at 10ºC. The interaction occurs with a Kd of 3.09 (±0.15) μM, at a stoichiometry of 0.97.

D) ITC 500μM of Hsp90-N injected into 50μM Rar1-CHORD$_{II}$ domain at 30ºC. The interaction occurs with a Kd of 22.3 (±2.4) μM, at a stoichiometry of 0.98.

E) Functional assay of Rar1 mutants (I153, E170 or W217) in *N*-mediated resistance against tobacco mosaic virus (TMV). *Myc-Rar1* or its derivatives are expressed in *N*-expressing *N. benthamiana* silenced for endogenous *Rar1*. Three days after the initial inoculation, GFP fused TMV is expressed by *Agrobacterium*. Myc-Rar1 (positive) and GUS (negative) were used as controls. Similar protein expression levels of Rar1 mutants were checked by western blotting using α-Myc antibody.

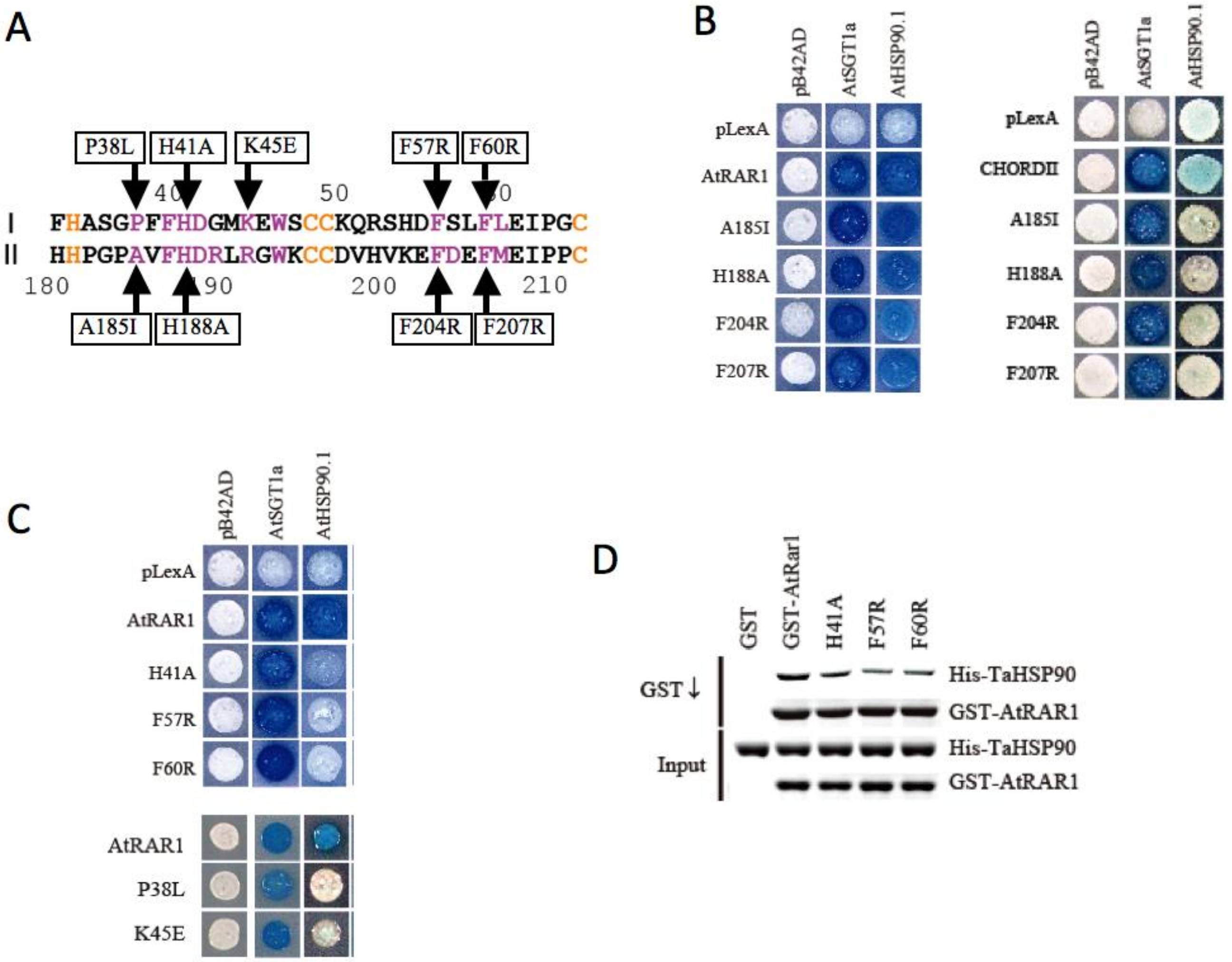

Figure S3 – (related to Figure 4) - Rar1 – Hsp90 interactions

A) Comparison of Hsp90-interacting residues in $CHORD_{I}$ and $CHORD_{II}$ domains. Residues involved in $CHORD_{II}$ interaction with Hsp90 from the structure described here, and their equivalents in the $CHORD_{I}$ domain, are shown in magenta. Zinc ligands are shown in gold. The positions of Rar1 mutants used in this figure are shown by arrows.

B) Yeast two-hybrid analysis of $CHORD_{II}$ mutants. Mutation of Rar1 residues A185, H188, F204 or F207 in the $CHORD_{II}$ domain only (left panel) but not in full length Rar1 (right panel) reduces the interaction with Hsp90.

C) Yeast two-hybrid analysis of $CHORD_{I}$ mutants. Mutation of Rar1 residues P38, H41, K45, F57, or F60 in the full length Rar1 reduces the interaction with Hsp90.

D) *In vitro* binding assays of Hsp90 with Rar1 derivatives. Mutation of Rar1 residues, H41, F57, or F60R reduces the interaction with Hsp90-N.

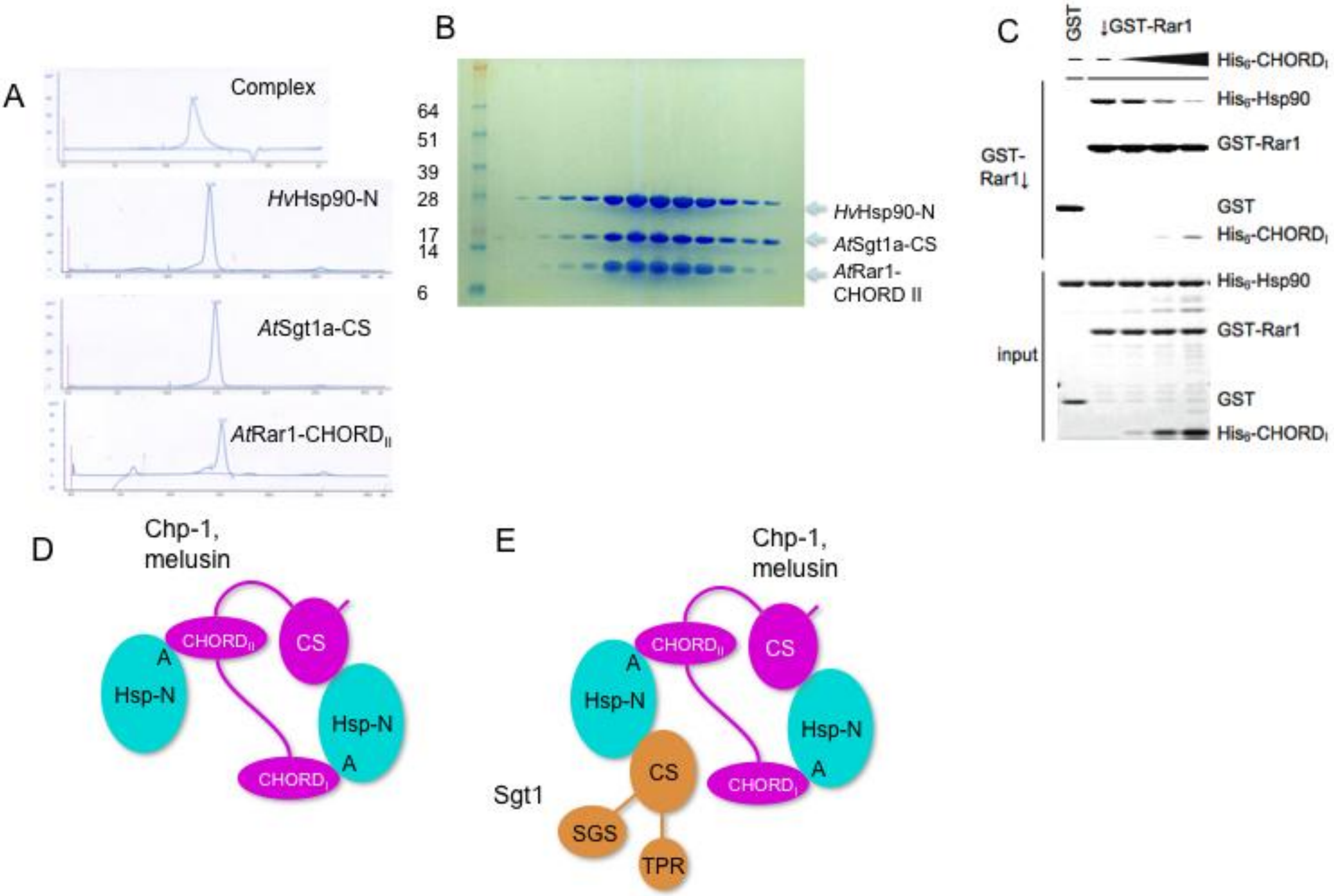

Figure S4 – (related to Figure 5) - CHORD – Sgt1 – Hsp90 complex

A) Gel filtration chromatography of the Rar1-CHORD$_{II}$ – Sgt1-CS – Hsp90-N complex and components. The experiment was carried out on a Superdex^{TM} 200 size exclusion column (Bed volume : 24 ml) pre-equilibrated with 20mM Tris pH 7.5, 150 mM NaCl and 50 µM $ZnCl_2$. For the complex, *Hv*Hsp90-N, *At*Sgt1a-CS and *At*Rar1-CHORD II were mixed at 1:1:1 molar ratio and adjusted to 200µl. Sample was injected and run through the column at the speed of 0.5 ml/min. 200µl of each of the isolated components with run separately. The mixture of the three clearly runs as a single peak eluting earlier on the column than any of the components, consistent with formation of a ternary complex.

B) SDS PAGE gel of the fractions across the peak of the gel-filtration profile of the complex. The three components are present at roughly stoichiometric equivalence, as judged by the intensity of the Coomasie staining.

C) Competition between CHORD$_I$ and Rar1 for binding to Hsp90 shows that a single Rar1 can simultaneously occupy both CHORD-domain binding sites in an Hsp90 dimer Methodology is essentially identical to that for FOIGURE 5e.

D) Model of the interaction of a mammalian CHORD protein with Hsp90. As with Rar1, each of the two CHORD-domains interacts with an N-terminal domain in the the Hsp90 dimer. The C-terminal CS domain of melusin/Chp-1, absent in the plant Rar1, interacts with one of the Hsp90-N domains in the same way as the separate CS domain of an Sgt1 protein.

E) as C), but an alternative model for a mammalian CHORD complex, in which an Sgt1 might also participate.

A

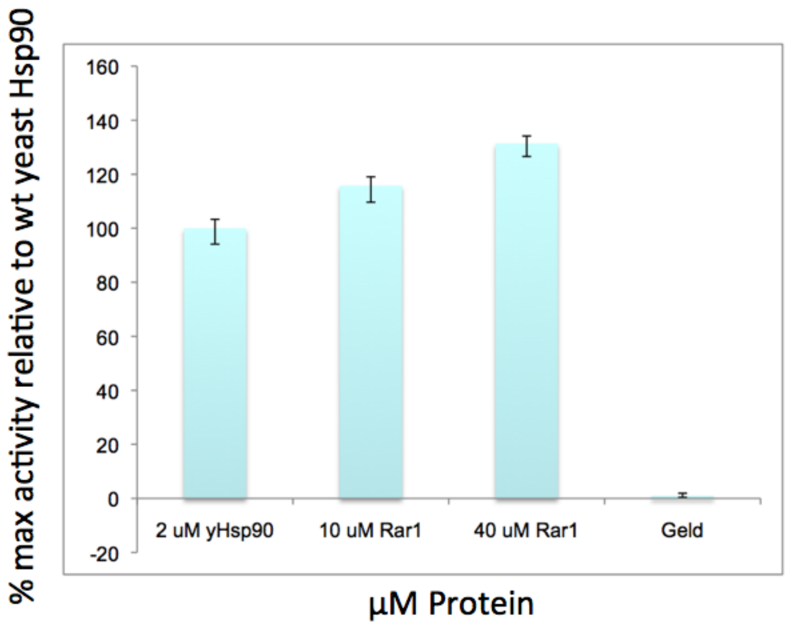

B

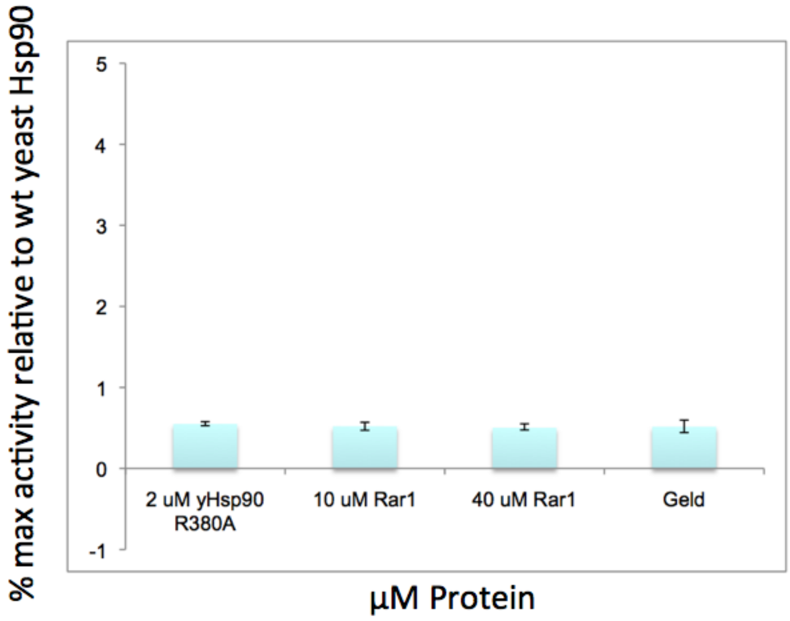

Figure S5 – (related to Figure 6) - Activation of the ATPase activity of yeast Hsp90 by Rar1.

A) Effect of Rar1 on the inherent ATPase of yeast Hsp90. Increasing concentrations of Rar1 promote and increase the ATP turnover of yeast Hsp90, achieving a 30% increase in activity at 20-fold excess. ATP hydrolysis is fully inhibited by the Hsp90-specific inhibitor geldanamycin (GD) demonstrating that the observed ATPase activity is a function of Hsp90 itself.

B) Effect of Rar1 on the inherent ATPase of yeast Hsp90-R380A catalytic-loop mutant. Increasing concentrations of Rar1 fail to activate the ATPase activity of the R380A mutant Hsp90, indicating that Arg 380 is essential for the Rar1 directed ATPase activation of Hsp90.
